# Supplementary material for: Pregnancy complications and cardiovascular disease risk perception: A qualitative study
Source: PLoS One. 2022 Jul 21;17(7):e0271722. doi: 10.1371/journal.pone.0271722 (PMC9302856; doi:10.1371/journal.pone.0271722)
Supplement: S1 File — (DOCX) [file pone.0271722.s001.docx]

**Interview Guide**

1. What are the complications that can occur during a pregnancy?
2. How common are these pregnancy complications?
3. What did your doctor/midwife tell you about pregnancy complications?
4. Did you read about pregnancy complications during your pregnancy?
   1. If Yes – where did you get this information from?
   2. If yes – was this information useful?
5. What information do you think women should get during pregnancy about these complications?
6. Do you think pregnancy complications have any effects after the pregnancy?
7. Is there a link between pregnancy complications and heart disease?
8. What time after pregnancy would a woman like to come back to hospital to attend a clinic?
9. Do you think women will attend a clinic after pregnancy if they were referred by their obstetrician?
10. What are the barriers to attending an appointment like this?
11. What sort of information will be useful for an appointment like this?
12. Will young women be interested in heart health immediately after pregnancy?
    1. If they say no – ask why not
13. What are the ways we can get women more interested in heart health at a young age?
    1. If not already mentioned – what would be a preferable structure, individual appointments or group sessions?
    2. Should these be tailored to specific pregnancy complications?
14. Do you think social media will help in keeping in touch with women about heart health?
    1. If yes – what social media platform would you prefer
15. Do you think young women will be happy to come for regular clinic visits to improve heart health starting after pregnancy?
16. Will women like to come to a clinic in hospital or go to their GP for heart health related matters?
    1. Depending on answer to above, why do you prefer GP/hospital clinic?
